# Label-Free Quantitative Proteomics Unravel the Impacts of Salt Stress on *Dendrobium huoshanense*

**DOI:** 10.3389/fpls.2022.874579

**Published:** 2022-05-12

**Authors:** Cheng Song, Yunpeng Zhang, Rui Chen, Fucheng Zhu, Peipei Wei, Haoyu Pan, Cunwu Chen, Jun Dai

**Affiliations:** ^1^College of Biological and Pharmaceutical Engineering, West Anhui University, Lu’an, China; ^2^Anhui Engineering Laboratory for Conservation and Sustainable Utilization of Traditional Chinese Medicine Resources, Lu’an, China; ^3^School of Life Sciences, East China Normal University, Shanghai, China

**Keywords:** salt stress, reactive oxygen species, carbon dioxide fixation, label-free quantitative proteomics, *Dendrobium*

## Abstract

Salt stress is a constraint on crop growth and productivity. When exposed to high salt stress, metabolic abnormalities that disrupt reactive oxygen species (ROS) homeostasis result in massive oxygen radical deposition. *Dendrobium huoshanense* is a perennial orchid herb that thrives in semi-shade conditions. Although lots of studies have been undertaken on abiotic stresses (high temperature, chilling, drought, etc.) of model plants, few studies were reported on the mechanism of salt stress in *D. huoshanense*. Using a label-free protein quantification method, a total of 2,002 differential expressed proteins were identified in *D. huoshanense*. The Kyoto Encyclopedia of Genes and Genomes (KEGG) enrichment indicated that proteins involved in vitamin B6 metabolism, photosynthesis, spliceosome, arginine biosynthesis, oxidative phosphorylation, and MAPK signaling were considerably enriched. Remarkably, six malate dehydrogenases (MDHs) were identified from deferentially expressed proteins. (NAD+)-dependent MDH may directly participate in the biosynthesis of malate in the nocturnal crassulacean acid metabolism (CAM) pathway. Additionally, peroxidases such as superoxide dismutase (SOD), peroxidase (POD), and catalase (CAT), as well as antioxidant enzymes involved in glutathione biosynthesis and some vitamins biosynthesis were also identified. Taken together, these results provide a solid foundation for the investigation of the mechanism of salt stress in *Dendrobium* spp.

## Introduction

Salt is a major factor impacting plants growth and output, among which sodium salt is the most frequently encountered hazard (Shrivastava and Kumar, 2015). Insufficient water and mineral nutrients cause plants to be somehow malnourished, which reduces the amount of chlorophyll and hence affects photosynthesis (Van Zelm et al., 2020). The effects of salt stress on photosynthesis often manifested in the chloroplasts (Yang Z. et al., 2020; Hameed et al., 2021). Through inhibiting the activities of phosphoenolpyruvate (PEP) and ribulose-1,5-bisphosphate carboxylase (Rubisco), heavy salt destabilized the chloroplast structure, which resulted in preventing the biosynthesis of chlorophyll and carotenoids, sealing the stomata, and reducing the photosynthetic rate (Hnilickova et al., 2021). Additionally, salt stress decreased chlorophyll biosynthesis and photosynthetic rate, and increased respiration rate and concentration of CO_2_ compensation point (Lei et al., 2018). Some studies suggested that salt stress even suppressed the overall electron transport chain (Lu and Vonshak, 2002; Sudhir et al., 2005).

Salt stress also impaired membrane permeability, malfunctioned several membrane-binding enzymes, and caused a range of metabolic abnormalities (Mansour, 2013; Yang W. et al., 2020; Hasanuzzaman et al., 2021; Ren et al., 2021). Ion and osmotic stress induced by salt stress result in a metabolic imbalance and the harmful deposition of ROS (Guo et al., 2019; Zhao et al., 2021). Antioxidant enzymes, such as superoxide Dismutase (SOD), catalase (CAT) and peroxidase (POD), as well as diverse antioxidants such as ascorbic acid (Vitamin C), glutathione (GSH), and carotenoids, etc., are reinforced under salt stress in plants (Akram et al., 2017; Sarker and Oba, 2018; Hasanuzzaman et al., 2020; Kumar et al., 2021). Numerous studies have reported that salt stress stimulates the activity of ROS scavenging enzymes and antioxidants (Choudhury et al., 2017; Huang et al., 2020). Salt stress induces ascorbic acid peroxidase and CAT, which improve tolerance to salinity and oxidative stress (Sofo et al., 2015). Overexpression of *OsAPXa/APXb* increased salt tolerance in rice, which shared a similar function with *OsGSTU4* (Lu et al., 2007; Sharma et al., 2014).

*Dendrobium huoshanense* is a semi-shade plant of the *Dendrobium* genus in the Orchidaceae family. Throughout the field cultivation, it is exposed to abiotic stresses such as high temperature, high salinity, and drought, all of which have a detrimental effect on its growth and biosynthesis of active components. *Dendrobium* orchids have adapted to high salinity situations by minimizing Na^+^ and Cl^–^ outflow to the shoot and maintaining a steady K^+^ afux level in their leaves. Heavy NaCl caused leaf water and osmotic potential (Abdullakasim et al., 2018). *Non-phosphorus glycerolipid synthase* (*NGLS*) genes are required for the homeostasis of membrane lipids under conditions of salt stress. It was indicated that *monogalactosyldiacylglycerol synthase 1* (*MGD1*) and (*sulfoquinovosyldiacylglycerol synthases*) *SQDs* were greatly induced by salt stress in *D. catenatum* leaves, while *digalactosyldiacylglycerol synthases* (*DGDs*) were primarily induced by salt stress in roots (Zhan et al., 2021). As a vital part of the antioxidant system, superoxide dismutase (SOD) is critical for protecting plants from oxidative stress. In *D. catenatum*, NaCl treatment greatly stimulated the expression of *Cu/ZnSODs* but reduced the expression of *FeSODs* (Huang et al., 2020).

To adapt to severe environment, plants holding the CAM pathway typically sealed their stomata during the day and open them at night (Töpfer et al., 2020). Some halophytes like *Peperomia*, *Portulaca*, and *Agropyron*, etc., altered their carbon assimilation involved in photosynthetic pathways from the C4 pathway to the CAM pathway (Bose et al., 2014; D’Andrea et al., 2014). *Dendrobium* species mostly feature CAM, which enables them to adapt to harsh environments. The expression profiles of *phosphoenolpyruvate carboxylase* (*PEPC*) and *phosphoenolpyruvate carboxylase kinase* (*PPCK*) in *Phalaenopsis* indicated that *PaPPCK* exhibits day/night fluctuation, implying that it may be involved in the regulation of CAM pathway (Ping et al., 2018). Some transcription factors, such as HD-Zip genes, are involved in the facultative CAM of *D. catenatum* under salt stress (Huang et al., 2021). In *D. nobile*, the WD40 repeat protein MS1 was downregulated by salt stress. Overexpression of *DnMSI1* in *Arabidopsis* resulted in decreased tolerance to salt stress (Cui et al., 2022). Polysaccharides have been shown to enhance plant resistance (Liu et al., 2019). GDP-mannose pyrophosphorylase (GMP) is involved in the biosynthesis of water-soluble polysaccharides in *D. officinale*. The *DoGMP1* transgenic *A. thaliana* showed better resistance to salt stress than the wild type (He et al., 2017). UDP glucose 4-epimerase (UGE) is also known for its role in the accumulation of water-soluble polysaccharide. Under salt stress, the *DoUGE* transgenic *A. thaliana* exhibited increased root length and fresh weight, as well as decreased proline and malondialdehyde production (Yu et al., 2017).

Although many studies have demonstrated the salt resistance of *Dendrobium* species, few have addressed the development of protein interaction networks and the systematic identification of antioxidant enzymes. To get a better understanding of the regulatory mechanism and salt-resistance mechanism of *D. huoshanense* under salt stress, total protein was isolated from high-sodium salt-treated and normally cultured *D. huoshanense* seedlings, respectively. A total of 2,002 proteins were identified, of which 1,088 were up-regulated and 418 were down-regulated. These deferential expressed proteins are mostly involved in photosynthesis, secondary metabolism, carbon assimilation, carbohydrate metabolism, and protein synthesis. Some differential expressed proteins were mainly involved in VB6 metabolism, photosynthesis, and antioxidant enzyme biosynthesis. These findings give us a scientific reference for the regulatory mechanism of salt tolerance in *Dendrobium* species.

## Materials and Methods

### Tissue Culture and NaCl Treatment Conditions

The tissue culture seedlings of *D. huoshanense* were obtained from Anhui Plant Cell Engineering Center in West Anhui University (Lu’an, China). The seeds were inoculated in December 2020, and subcultures were undertaken every 40 days. Murashige and Skoog medium (including 1.5% agar and 30 g/L sucrose) was used as the culture medium, with no hormones added. The grown conditions were set at a 25 ± 2°C temperature, and a 12-h light/dark cycle. The air humidity in the tissue culture room is about 60%. For salt stress experiment, tissue culture seedlings from more than five generations were chosen. The samples in the control group were sprayed with the same amount of water daily, and the samples in the NaCl group were first sprayed with a solution containing 50 mM of NaCl. An increase of 50 mM NaCl was made every other day in the experimental group, and the concentration was eventually brought up to and maintained at 250 mM for 7 days. Then, the second and third leaves of annual leaves were selected for three biological replicates.

### Preparation and Determination of Total Protein

The stored samples were taken from the −80°C refrigerator, then transferred to a pre-cooled tube mixed with SDT protein lysis buffer (4% SDS, 10 mM DTT, 100 mM TEAB). The lysate was reacted to at 95°C for 8 min, then centrifuged at 12,000 *g* for 15 min. The supernatant was collected and mixed with 10 mM DTT to react at 56°C for 1 h, followed by IAM to react at room temperature for 1 h in the dark. The solution was precipitated at −20°C for at least 2 h, then centrifuged at 12,000 *g* for 15 min. To resuspend the supernatant, 1 mL of −20°C pre-cooled acetone was added, and the pellet was centrifuged at 12,000 *g* for 15 min. A sufficient amount of protein solubilizer (8 M urea, 100 mM TEAB, pH = 8.5) was supplied to dissolve the protein precipitation (Wiśniewski et al., 2009; Wu et al., 2014).

Using the Bradford protein quantification kit, BSA standard protein solutions with different concentration and test sample solutions with various dilution ratios were added to the 96-well plate. Each gradient was repeated three times. As soon as the 180 L of G250 staining solution has been added, the absorbance should be measured at 595 nm. The protein concentration of the sample to be tested was calculated based on the standard curve and the absorbance of the standard protein solution.

### Protein Proteolysis and Liquid Chromatography/Mass Spectrometry Analysis

Protein samples were treated with 100 μL of denaturing solution (8 M urea, 100 mM TEAB, pH = 8.5). Trypsin and 100 mM of TEAB buffer were put into the samples and digested for 4 h at 37°C. The digestion solution was continuously mixed with trypsin and CaCl_2_ solution overnight. The reaction solution was adjusted to a pH of less than 3 with formic acid and centrifuged at 12,000 *g* for 5 min. Desalting protein was obtained by slowly passing through a C18 column. After that, the supernatant was rinsed three times with the washing solution (containing 0.1% formic acid, 3.0% acetonitrile). The filtrate was collected and lyophilized after the supernatant was diluted with an appropriate amount of eluent (70% acetonitrile, containing 0.1% formic acid). Mobile phase solutions A (100% water, containing 0.1% formic acid) and B (80% acetonitrile, containing 0.1% formic acid) were prepared. To dissolve the lyophilized powder, 10 μL of solution A was mixed and centrifuged at 14,000 *g* for 20 min at 4°C.

For LC/MS analysis, the EASY-nLC 1,200 nanoscale ultrahigh performance liquid chromatography equipment was used for the chromatographic analysis. The guard column (4.5 cm × 75 m, 3 μm) and analytical column (15 cm × 150 μm, 1.9 μm) were constructed, respectively. A Q Exactive HF-X mass spectrometer equipped with a Nanospray FlexTM (ESI) ion source was used for mass spectrometry. The voltage of the ion spray was set to 2.1 kV, and the temperature of ion transfer tube was set to 320°C. The mass spectrum was obtained in a data-dependent mode, using a full scan range of m/z 350–1,500. The resolution of the main mass spectrometer was set at 60,000. The injection time was a maximum of 20 ms. Precursor ions with an ionic strength of TOP 40 were selected and fragmented for MS^2^ detection in a high energy induced dissociation (HCD) mode. The secondary mass spectrometry resolution was set at 15,000 and the maximum injection time was 45 ms. The peptide fragmentation collision energy was 27%, and the dynamic exclusion period was 20 s.

### Qualitative and Quantitative Analysis of Differential Proteins

The quantification of proteins was carried out using Proteome Discoverer 2.2 (Thermo Fisher Scientific, United States). The protein sequence of *D. huoshanense* was retrieved through a reference genome and acquired from the NCBI (Bioproject Number: PRJNA597621). The library search parameters were set as follows: a mass tolerance of 10 ppm was used for precursor ions, and a mass tolerance of 0.02 Da was used for fragment ions. To further narrow down the search results, we used the following options: peptide spectrum matched with a reliability of more than 99% are trusted PSMs. Proteins containing at least one unique peptide (unique peptide) are trusted proteins, and only trusted spectrum peptides and proteins are retained. Additionally, false discovery rate (FDR) was performed to eliminate peptides and proteins, which was greater than 1%. Statistical analysis was performed on the protein quantification results by the *T*-test test, and the differential expressed proteins between the experimental group and the control group were screened. The raw proteomics data have been deposited to the ProteomeXchange Consortium^1^ via the iProX partner repository with the dataset identifier PXD031654 (Ma et al., 2019). Proteins with an fold change ≥ 4.0 and a *P*-value ≤ 0.05 were defined as up-regulated, while proteins with an fold change ≤ 0.25 and a *P*-value ≤ 0.05 were defined as down-regulated. InterProScan software was used for GO and IPR functional annotation, while COG and KEGG databases were used for functional protein family and pathway analysis (Jones et al., 2014). The STRING software was used to predict potential protein-protein interactions (Franceschini et al., 2013). The visualized results from volcano plots, heatmaps, PCA, GO, KEGG, etc., were obtained using the R script.

## Results

### Identification and Functional Annotation of Proteins

Over 63,000 matched spectra were acquired in this experiment, along with 12,402 peptides and 2,325 proteins. Among them, the control group identified an average of 1,695 proteins, while the high sodium salt treatment group identified an average of 2,182 proteins (Supplementary Figure 1A). The length of the peptide segment is primarily between 7 and 25 amino acids (Supplementary Figure 1B). The peptide coverage of 81.38% of proteins covered less than 30% of the detected proteins, indicating that the depth of protein identification still needs to be improved (Supplementary Figure 1C). The molecular weight distribution of the identified proteins suggested that they were mostly in the range of 10–60 kDa (Supplementary Figure 1D). By calculating the mass error distribution of the precursor ions, it was indicated that over 90% of proteins had mass errors of less than 0.00392 Da (Supplementary Figure 1E). Principal component analysis suggested that the control and NaCl treatment groups had significant differences in the expression levels (Supplementary Figure 1F).

**FIGURE 1:**
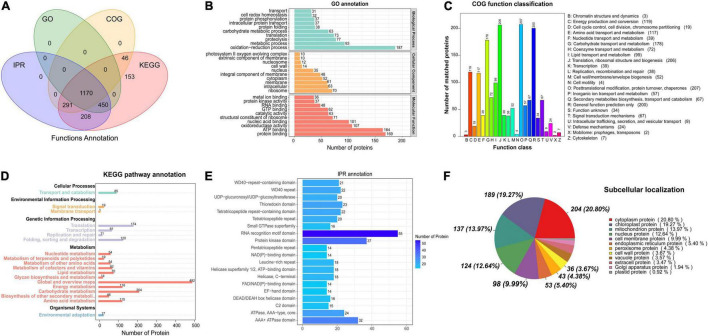
Functional annotation of the proteins based on database searches. **(A)** Venn diagram of proteins annotated in different databases. **(B)** GO functional annotation. **(C)** COG functional classification. **(D)** KEGG pathway annotation. **(E)** IPR protein domain annotation. **(F)** Analysis of protein subcellular localization.

GO, KEGG, COG, and other databases were used to annotate the functions of all the identified proteins (Supplementary Table 1). InterProScan was used to analyze the gene family and structure of all detected proteins by using Pfam, PRINTS, ProDom, SMART, ProSite, and PANTHER. A total of 1,170 proteins were annotated by the GO, KEGG, COG, and IPR (Figure 1A). Based on the GO functional annotation, the proteins were mainly involved in redox, metabolism, proteolysis, translation, and carbohydrate metabolism. Some proteins provide various biological functions, including protein binding, ATP binding, nucleic acid binding, oxidoreductase activity, and structural components of the ribosome (Figure 1B). The COG annotation indicated that the highest number of homologous genes were found in translation, ribosomal structure and biogenesis, posttranslational modification, protein turnover, chaperones, glucose transport and metabolism (Figure 1C). The KEGG pathway annotation indicated that the major metabolic pathways were glucose metabolism, energy metabolism, amino acid metabolism, protein transport and catabolism, and translation activities (Figure 1D). IPR annotation indicated that all identified proteins contained a huge number of RNA recognition motif domains, protein kinase domains, and ATPase domains (Figure 1E). Subcellular localization analysis showed that 20.8% of proteins were located in the cytoplasm, 19.2% in the chloroplast, 13.9% in the mitochondria, and 12.6% in the nucleus (Figure 1F).

### Quantification and Expression Profiles of Differential Protein

**FIGURE 2:**
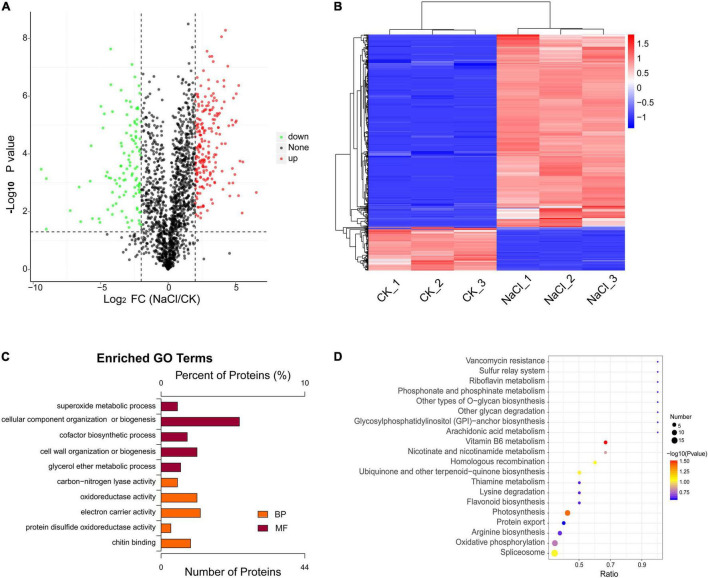
Differential analysis and functional enrichment of the proteins under salt treatment. **(A)** Differential protein volcano plot. **(B)** Differential protein cluster analysis plot. **(C)** GO enrichment analysis of differential proteins. **(D)** KEGG enrichment analysis of differential proteins.

A total of 663 differentially expressed proteins were identified, 541 of which were significantly differentially up-regulated proteins (fold-change > 4, *p* < 0.05). There were 122 proteins that were significantly down-regulated (fold-change < 0.25, *p* < 0.05). The volcano plot displays the distribution of differential proteins between the two groups (Figure 2A). Hierarchical clustering analysis indicated that salt stress increased the expression of most differential proteins (Figure 2B). These proteins were mostly involved in the organization or biogenesis of cellular components, the biosynthesis of cofactors, the activity of oxidoreductases, and the activity of electron carriers (Figure 2C). Vitamin B6 metabolism, photosynthesis, spliceosome, arginine biosynthesis, oxidative phosphorylation, and MAPK signaling pathways were highly enriched (Figure 2D). We investigated the major pathways enriched in KEGG and discovered that salt stress affected *D. huoshanense* primarily by altering photosynthesis intensity, oxidative phosphorylation, and carbon fixation processes. A total of 41 proteins were identified involved in photosynthesis, thirteen of which were up-regulated under salt stress, including plastocyanin, photosystem II, and the cytochrome b6-f complex. Thirty-four proteins participated in oxidative phosphorylation, thirteen of which were upregulated. These proteins mainly included V-type ATPase, F-type ATPase, NADH dehydrogenase 1, cytochrome c reductase, and succinate dehydrogenase (Table 1). Through oxidative phosphorylation, salt stress elevates the expression of photosystem II and critical electron transport chain regulators, as well as the production of ATP for energy supply and carbon assimilation.

**TABLE 1:** Differentially expressed proteins involved in photosynthesis and oxidative phosphorylation.

KEGG term	Protein ID	Fold change	*p*-value	Up or downregulated	KEGG description
Photosynthesis	Dhu000016663	Infinity	0.00	Up	Plastocyanin
	Dhu000022000	4.43 ± 2.21	2.27E-07	Up	Photosystem II oxygen-evolving enhancer protein 2
	Dhu000018784	10.17 ± 4.37	1.45E-06	Up	Cytochrome b6-f complex iron-sulfur subunit
	Dhu000002825	7.16 ± 4.92	2.18E-06	Up	Photosystem II psb27 protein
	Dhu000002238	27.70 ± 3.08	1.00E-05	Up	Cytochrome b6-f complex iron-sulfur subunit
	Dhu000019960	4.02 ± 0.56	2.56E-05	Up	PsbP family
	Dhu000002675	6.57 ± 4.33	3.55E-05	Up	F-type h + -transporting ATPase subunit β
	Dhu000022570	5.83 ± 4.52	1.04E-04	Up	F-type h + -transporting ATPase subunit b
	Dhu000027584	7.20 ± 3.26	3.70E-04	Up	Photosystem II oxygen-evolving enhancer protein 3
	Dhu000018717	8.68 ± 10.79	4.43E-04	Up	F-type h + -transporting ATPase subunit δ
	Dhu000028844	30.72 ± 11.10	5.18E-04	Up	Ferredoxin
	Dhu000028842	9.22 ± 12.75	3.82E-03	Up	Ferredoxin
Oxidative phosphorylation	Dhu000008527	Infinity	0.00	Up	V-type h + -transporting ATPase 16 kda proteolipid subunit
	Dhu000010659	Infinity	0.00	Up	Nadh dehydrogenase (ubiquinone) 1 beta subcomplex subunit 7
	Dhu000028439	Infinity	0.00	Up	Nadh dehydrogenase (ubiquinone) fe-s protein 6
	Dhu000006846	5.22 ± 4.21	6.68E-06	Up	F-type h + -transporting ATPase subunit d
	Dhu000014507	31.93 ± 2.36	8.05E-06	Up	Nadh dehydrogenase (ubiquinone) 1 α subcomplex subunit 5
	Dhu000002675	6.57 ± 4.33	3.55E-05	Up	F-type h + -transporting ATPase subunit β
	Dhu000000294	12.20 ± 0.79	6.18E-05	Up	Nadh dehydrogenase (ubiquinone) 1 β subcomplex subunit 9
	Dhu000022570	5.83 ± 4.52	1.04E-04	Up	F-type h + -transporting ATPase subunit b
	Dhu000006207	7.82 ± 1.24	1.43E-04	Up	Ubiquinol-cytochrome c reductase cytochrome c1 subunit
	Dhu000018717	8.68 ± 10.79	4.43E-04	Up	F-type h + -transporting ATPase subunit δ
	Dhu000000527	8.79 ± 0.37	4.84E-04	Up	Succinate dehydrogenase (ubiquinone) iron-sulfur subunit
	Dhu000017283	92.41 ± 19.13	2.16E-03	Up	V-type h + -transporting ATPase subunit g
	Dhu000014683	4.47 ± 0.69	2.23E-03	Up	Succinate dehydrogenase (ubiquinone) flavoprotein subunit

**FIGURE 3:**
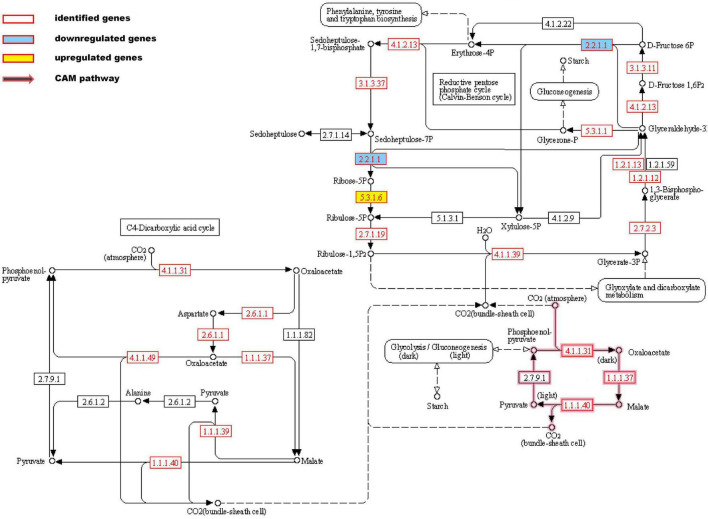
The key genes involved in carbon fixation based on KEGG enriched pathways.

Carbon assimilation is a process of converting CO_2_ into carbohydrates. Thirty-one proteins were involved in the carbon fixation pathway, including those involved in the Calvin cycle, the CAM pathway, and the C_4_ cycle (Figure 3). Salt stress increased the expression of ribose 5-phosphate isomerase and decreased the expression of transketolase. There were two phosphoenolpyruvate carboxylases (PEPC) identified. We hypothesize that they are involved in the formation of oxaloacetate in the C_4_ cycle and the CAM pathway, respectively. Malate dehydrogenases (MDHs) catalyze the conversion of malate to oxaloacetate or pyruvate by taking NAD(H) or NADP(H) as cofactors. Six malate dehydrogenase family proteins were identified in this study, three of which were (NAD^+^)-dependent MDH, one of which was (NADP^+^)-dependent MDH (oxaloacetate-decarboxylating), and two of which were (NAD^+^)-dependent MDH (decarboxylating).

Besides the photosystem proteins, many antioxidant enzymes that scavenge oxygen free radicals were identified, which included SOD, POD, and CAT (Table 2). Three SODs, eleven PODs and two CATs were identified. Except for one peroxidase, one L-ascorbate peroxidase, one glutathione peroxidase, and one CAT, all of the SODs were significantly upregulated. Salt stress also stimulated the biosynthesis of antioxidants such as vitamin B6/B1, vitamin C, and glutathione in *D. huoshanense*. Salt stress increased the expression of *pyridoxal 5′-phosphate synthase* (*PDX1*) and directly increased the content of pyridoxal 5-phosphate (PLP). However, it decreased the expression of *pyridoxine 4-dehydrogenase* (*PLR1*), thereby impairing pyridoxal biosynthesis. Vitamin C is a powerful natural antioxidant, and salinity (mostly NaCl) could promote the production of root apoplastic ascorbyl radicals (Makavitskaya et al., 2018). Here, fifteen ascorbate-related metabolic enzymes were identified, three of which were upregulated and one of which was downregulated. Under salt stress, the expression of the dehydroascorbic reductase rose approximately 12-fold. A total of 21 key enzymes in glutathione biosynthesis were identified, including 6-phosphogluconate dehydrogenase, glutathione S-transferase, glutathione peroxidase, pyruvate/2-oxoglutarate dehydrogenase, glutathione synthase, NADP-dependent isocitrate dehydrogenase, ascorbate peroxidase, and leucyl aminopeptidase. Among them, four glutathione S-transferases could be induced by ROS and one glutathione peroxidase was upregulated. Eight ubiquinone biosynthetic genes were identified in the study, three of which were NAD/NADP-dependent quinone dehydrogenases, and one of which was ubiquinone monooxygenase. Additionally, three genes involved in NAD biosynthesis were identified, among which phosphodiesterase was upregulated, while NAD synthetase was downregulated under salt stress.

**TABLE 2:** Peroxidase and key enzyme of antioxidant biosynthesis involved in ROS scavenging.

KEGG term	Protein ID	Fold change	*p*-value	Up or downregulated	KEGG description
Peroxidases	Dhu000014602	5.03 ± 0.08	1.74E-05	Up	Catalase
	Dhu000028022	4.54 ± 1.57	8.58E-07	Up	Superoxide dismutase, cu-zn family
	Dhu000015323	10.10 ± 17.45	4.02E-04	Up	Superoxide dismutase, cu-zn family
	Dhu000016068	Infinity	0.00	Up	Superoxide dismutase, cu-zn family
	Dhu000005526	Infinity	0.00	Up	Peroxidase
	Dhu000018823	10.11 ± 3.15	6.37E-08	Up	L-ascorbate peroxidase
	Dhu000018044	6.13 ± 2.97	1.31E-07	Up	L-ascorbate peroxidase
	Dhu000010171	6.78 ± 1.23	8.26E-04	Up	Glutathione peroxidase
	Dhu000028960	0.03 ± 0.01	1.76E-02	Down	L-ascorbate peroxidase
Vitamin B6 metabolism	Dhu000007824	5.97 ± 0.14	6.68E-03	Up	Pyridoxal 5′-phosphate synthase
	Dhu000011167	2.27 ± 0.02	3.15E-02	None	Pyridoxamine 5′-phosphate oxidase
	Dhu000002640	Infinity	0.00	Up	Pyridoxal 5′-phosphate synthase
	Dhu000022208	0.57 ± 0.06	3.72E-01	None	Phosphoserine aminotransferase
	Dhu000028434	0.01 ± 0.01	9.17E-03	Down	Pyridoxine 4-dehydrogenase
	Dhu000010367	Infinity	0.00	Up	Phosphoserine aminotransferase
Thiamine metabolism	Dhu000026717	6.44 ± 0.51	1.83E-04	Up	Cysteine desulfurase
	Dhu000008600	4.59 ± 0.67	6.99E-03	Up	Adenylate kinase
Ascorbate and aldarate metabolism	Dhu000017762	11.96 ± 0.69	1.42E-06	Up	Dehydroascorbic reductase
	Dhu000018823	10.11 ± 3.15	6.37E-08	Up	L-ascorbate peroxidase
	Dhu000018044	6.13 ± 2.97	1.31E-07	Up	L-ascorbate peroxidase
	Dhu000028960	0.03 ± 0.01	1.76E-02	Down	L-ascorbate peroxidase
Glutathione metabolism	Dhu000018044	6.13 ± 2.97	1.31E-07	Up	L-ascorbate peroxidase
	Dhu000017762	11.96 ± 0.69	1.42E-06	Up	Dehydroascorbic reductase
	Dhu000023012	4.58 ± 1.01	2.34E-03	Up	Glutathione S-transferase
	Dhu000018823	10.11 ± 3.15	6.37E-08	Up	L-ascorbate peroxidase
	Dhu000010171	6.78 ± 1.23	8.26E-04	Up	Glutathione peroxidase
	Dhu000014453	4.62 ± 0.14	1.42E-02	Up	Glutathione S-transferase
	Dhu000020272	6.32 ± 0.38	2.83E-04	Up	Glutathione S-transferase
	Dhu000028960	0.03 ± 0.01	1.76E-02	Down	L-ascorbate peroxidase

### The Primary and Secondary Metabolic Enzymes of *Dendrobium huoshanense* Under Salt Stress

**FIGURE 4:**
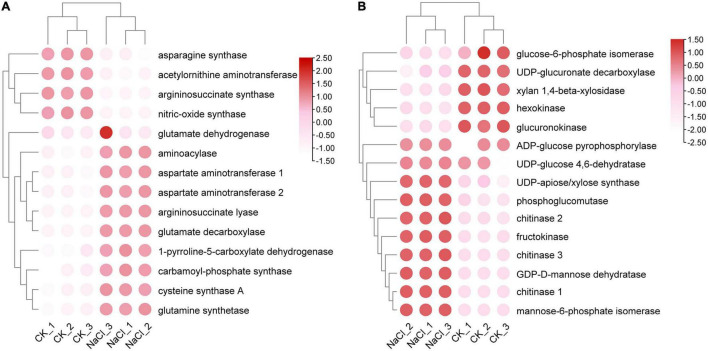
Analysis of differential expression patterns of amino acid and glucose metabolism under salt stress. **(A)** Expression profile of amino acid metabolism related genes. **(B)** Expression profile of glucose metabolism related genes.

Salt stress affects a number of enzymes involved in amino acid biosynthesis and sugar metabolism. A total of 14 proteins were involved in amino acid biosynthesis, of which cystine synthase A, argininosuccinate lyase, glutamate decarboxylase, and aminoacylase were up-regulated under salt stress. The expressions of asparagine synthase, argininosuccinate synthase, acetylornithine aminotransferase, and nitric-oxide synthase were reduced (Figure 4A). Fifteen enzymes involved in the metabolism of amino sugars and nucleoside sugars were identified. The expressions of chitinases, fructokinases, UDP-xylose synthase, GDP-D-mannose dehydratase, and mannose-6-phosphate isomerase were significantly up-regulated, while the expression of hexokinase was significantly downregulated (Figure 4B). The results suggested that salt stress exacerbated polysaccharide degradation and monosaccharide conversion. Salt stress suppressed the expression of chalcone isomerase but enhanced the expression of chalcone synthase.

**FIGURE 5:**
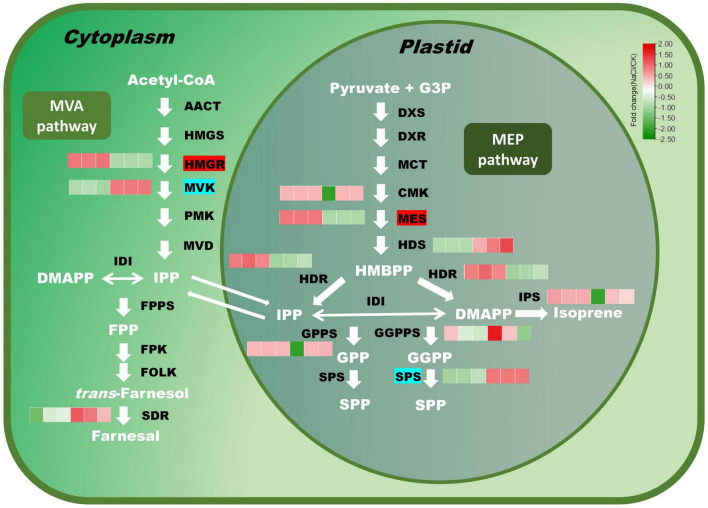
Expression profiles of key genes in the terpenoid biosynthesis pathway under salt stress. Red modules denote upregulation, while green modules denote downregulation. Genes with a red background have significantly higher expression, while genes with an indigo background have significantly lower expression.

Terpenoids are a broad class of natural compounds composed of isoprene units. Twelve terpene backbone biosynthesis genes were identified in this study. Indoleglycerol phosphate is the precursor of indole diterpene alkaloid. The additional eleven genes were mainly involved in monoterpene, sesquiterpene, diterpene, and polyterpene biosynthesis (Figure 5). Under salt stress, 2C-methyl-D-erythritol 2,4-cyclodiphosphate synthase (MES) and hydroxymethylglutaryl-CoA reductase (HMGR) were significantly enhanced, whereas MVK (mevalonate kinase) and SPS (solanesyl diphosphate synthase) were dramatically decreased. The expression of NADP-dependent farnesol dehydrogenase (SDR) dropped under salt stress. HDS catalyzes the conversion of 2-C-methyl-D-erythritol 2,4-cyclodiphosphate (MEP) to 4-hydroxy-3-methyl-but-2-enyl diphosphate (HMBPP) in the MEP pathway. HDR promoted the transformation of HMBPP into IPP and DMAPP, respectively. The expression of *HDR* was induced under salt stress, whereas the expression of *HDS* was reduced. GPPS and GGPPS are responsible for the biosynthesis of monoterpenes and diterpenoids. There was no significant difference in the expression level of GPPS under salt stress, while the expression level of GGPPS fluctuated somewhat across samples.

### The Differential Protein Interaction Network

**FIGURE 6:**
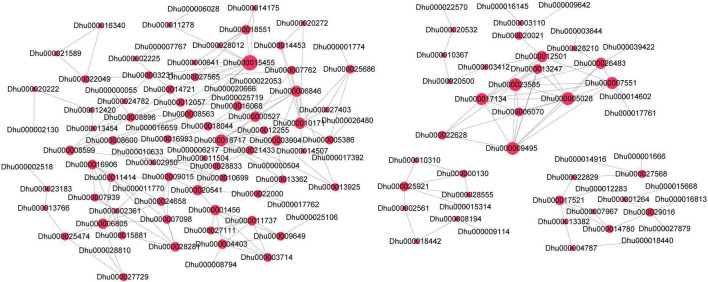
The differential protein interaction network. Node size indicates the number of the interacting proteins. The red color represents significantly positive expression.

The differential expressed proteins were screened out from 2,002 proteins to construct a protein-protein interaction network. Totally, 281 pairs of differential proteins with a score greater than 600 were present (Figure 6). Dhu000016068 (SOD) had a strong interaction with Dhu000020666 (copper chaperone for superoxide dismutase). Dhu000008599 (CTP synthase) exhibited a strong interaction with Dhu000008563 (nucleoside-diphosphate kinase). Dhu000018717 (F-type ATPase subunit δ) strongly interacted with Dhu000006846 (F-type ATPase subunit d). Dhu000015455 (3-hydroxyacyl-CoA dehydrogenase) interacted with Dhu000018551 (carnitine racemase) and Dhu000014175 (Electron transfer flavoprotein subunit β), respectively. Among the differential downregulated proteins, Dhu000002663 (transketolase) interacted strongly with the up-regulated protein Dhu000022049 (transaldolase). Dhu000017049 (NAD + synthetase) had a significant interaction with Dhu000025116 (agmatine deiminase).

## Discussion

Salt stress has a detrimental effect on almost essential life processes of plants, such as photosynthesis, protein synthesis, energy metabolism, and material metabolism (Zhang et al., 2021). To combat daytime water loss, some C_3_ plants evolved the CAM pathway. *Dendrobium* is a perennial semi-shade herb, and some varieties are facultative CAM plants (Cheng et al., 2019; Li et al., 2019). In nocturnal cells, phosphoenolpyruvate (PEP) works as a CO_2_ acceptor, which is catalyzed by PEP carboxylase to generate oxaloacetate. Malate is carried from the vacuole to the chloroplast for decarboxylation at the daytime, resulting in the release of CO_2_. The CAM pathway allows *Dendrobium* plants to survive and flourish in hostile environments. A dozen genes involved in photosynthesis were screened out in the study. Ferredoxin and the iron-sulfur subunit of the cytochrome b6-f complex showed the highest levels of expression after salt treatment, which were 30-fold and 27-fold greater than those in the control group, respectively. Additionally, salt stress greatly enhanced the expression of proteins involved in photosystem II and F-type ATPases.

Malate metabolism is altered when exposed to high salinity. To maintain plant growth and stress tolerance, malate dehydrogenases (MDHs) catalyze the reversible reaction between malate and oxaloacetic acid (Tomaz et al., 2010; Zhao et al., 2020). NAD-dependent MDH negatively regulated salt stress-mediated vitamin B6 biosynthesis (Nan et al., 2020). Compared with wild-type rice, the content of pyridoxine was significantly reduced in *OsMDH1*-overexpressed plants. In the chloroplast, NAD-dependent MDH is responsible for the conversion of oxaloacetate to malate, while NADP-dependent MDH is responsible for the conversion of malate to pyruvate and the release of a portion of carbon dioxide (Schreier et al., 2018; Rao and Dixon, 2019). (NADP+)-specific MDH decarboxylates delivered malate into chloroplasts, and the released CO_2_ enters the Calvin cycle for fixation. In this study, the expression of the (NAD+)-dependent MDHs were 2.1, 2.3, and 1.9 fold higher than those in the control group, respectively. However, salt stress had no effect on the expression of the NADP-dependent MDH (oxaloacetate-decarboxylating), and the underlying explanation and molecular control mechanism remain unresolved.

Several MAPK signaling proteins were identified in this study, such as serine/threonine protein kinase SRK2 (SnRK2), NDPK2, MKK4/5, etc. Enormous H_2_O_2_ further cascades MAPK signaling, thereby intensifying the transduction of downstream signals and the production of peroxidase (Schieber and Chandel, 2014). Salt treatment also induced the biosynthesis of antioxidants such as glutathione, VB6, VB1, and VC. Massive free radicals (especially hydrogen peroxide) and MAPK signal transduction may be the reasons for the formation of free radical scavenging enzymes and antioxidants in *D. huoshanense*. *SOS4* encodes a pyridoxine kinase that catalyzes the conversion of vitamin B6 to pyridoxal phosphate (Rueschhoff et al., 2013; Mangel et al., 2019). The *sos4* mutant accumulated more sodium ions and fewer potassium ions than the wild type under salt stress. Our results showed that salt stress caused a significant decrease in the expression of *DhPLR1*, but a significant increase in the expression of *DhPDX1*. The expression of *pyridoxamine 5′-phosphate oxidase* (*PDX3*) was not significantly increased. Glutathione peroxidase is responsible for the conversion between glutathione disulfide (GSSH) and glutathione (GSH) (Kerksick and Willoughby, 2005; Hasanuzzaman et al., 2019). The expression of glutathione peroxidase increased 12-fold after salt treatment.

Salt stress not only inhibits plant development but also stimulates the expression of relevant genes of secondary metabolic pathways (Yang and Guo, 2018; Chen et al., 2019; Valifard et al., 2019; Patel et al., 2020; Xu et al., 2020). Eleven terpenoid biosynthetic genes were identified, the bulk of which were involved in monoterpene, sesquiterpene, and diterpene biosynthesis. Salt stress significantly induced the expression of *HMGR*. Taking HMAPP as substrate, HDR produced DMAPP and IPP, respectively. The expression of *HDR* was induced by salt stress, while the expression of *HDS* was suppressed. GPPS and GGPPS are responsible for the biosynthesis of monoterpenoids and diterpenoids. There was no significant difference in the expression of *GPPS* under salt stress, while the expression of *GGPPS* was greatly affected by salt stress.

## Conclusion

In this study, we employed a label-free protein quantification approach to analyze the differentially expressed proteins under salt stress. A total of 663 differential genes were screened, 541 of which were significantly up-regulated and 122 of which were down-regulated. Carbohydrate metabolism, energy metabolism, amino acid metabolism, protein transport and catabolism, and translation processes were susceptible to salt stress. Salt stress also affected photosynthesis, oxidative phosphorylation, and carbon fixation processes to adjust for salt stress. A total of 31 proteins involved in carbon fixation were identified, including those participated in Calvin cycle, CAM pathway, and the C_4_ cycle. The MDHs was demonstrated to be involved in the carbon assimilation in C_4_ and CAM pathway. A series of peroxidases involved in scavenging oxygen free radicals were identified. Salt stress enhanced the formulation of antioxidants such as vitamins and glutathione in *D. huoshanense*. Study on the regulation mechanism of salt stress in *D. huoshanense* is lacking, our results provide a scientific basis for the screening of salt tolerance genes and the improvement of *Dendrobium* species.

## Data Availability Statement

The datasets presented in this study can be found in online repositories. The names of the repository/repositories and accession number(s) can be found in the article/Supplementary Material.

## Author Contributions

CS, YZ, and JD discussed the writing plan and edited the manuscript. CS and YZ drafted the manuscript. RC, FZ, PW, HP, and CC conducted the experiment. CS, FZ, and JD acquired the funding. All authors have read, reviewed, and approved the submitted version.

## Conflict of Interest

The authors declare that the research was conducted in the absence of any commercial or financial relationships that could be construed as a potential conflict of interest.

## Publisher’s Note

All claims expressed in this article are solely those of the authors and do not necessarily represent those of their affiliated organizations, or those of the publisher, the editors and the reviewers. Any product that may be evaluated in this article, or claim that may be made by its manufacturer, is not guaranteed or endorsed by the publisher.

## Footnotes

1
http://proteomecentral.proteomexchange.org

## Supplementary Material

Supplementary Figure 1 Data quality assessment and protein identification. **(A)** Number of proteins identified in control and NaCl groups. **(B)** Peptide length distribution and number. **(C)** Protein coverage distribution and quantity. **(D)** Protein molecular weight distribution and quantity. **(E)** Precursor ion mass tolerance distribution. **(F)** PCA analysis of control group and NaCl treatment group.

Supplementary Table 1 The TPM values and annotations of differentially expressed proteins between CK and NaCl treatment groups.

Supplementary Table 2 Score value of protein-protein interaction.
