# Supplementary figures and images for: Label-Free Quantitative Proteomics Unravel the Impacts of Salt Stress on Dendrobium huoshanense
Source: Front Plant Sci. 2022 May 12;13:874579. doi: 10.3389/fpls.2022.874579 (PMC9134114; doi:10.3389/fpls.2022.874579)

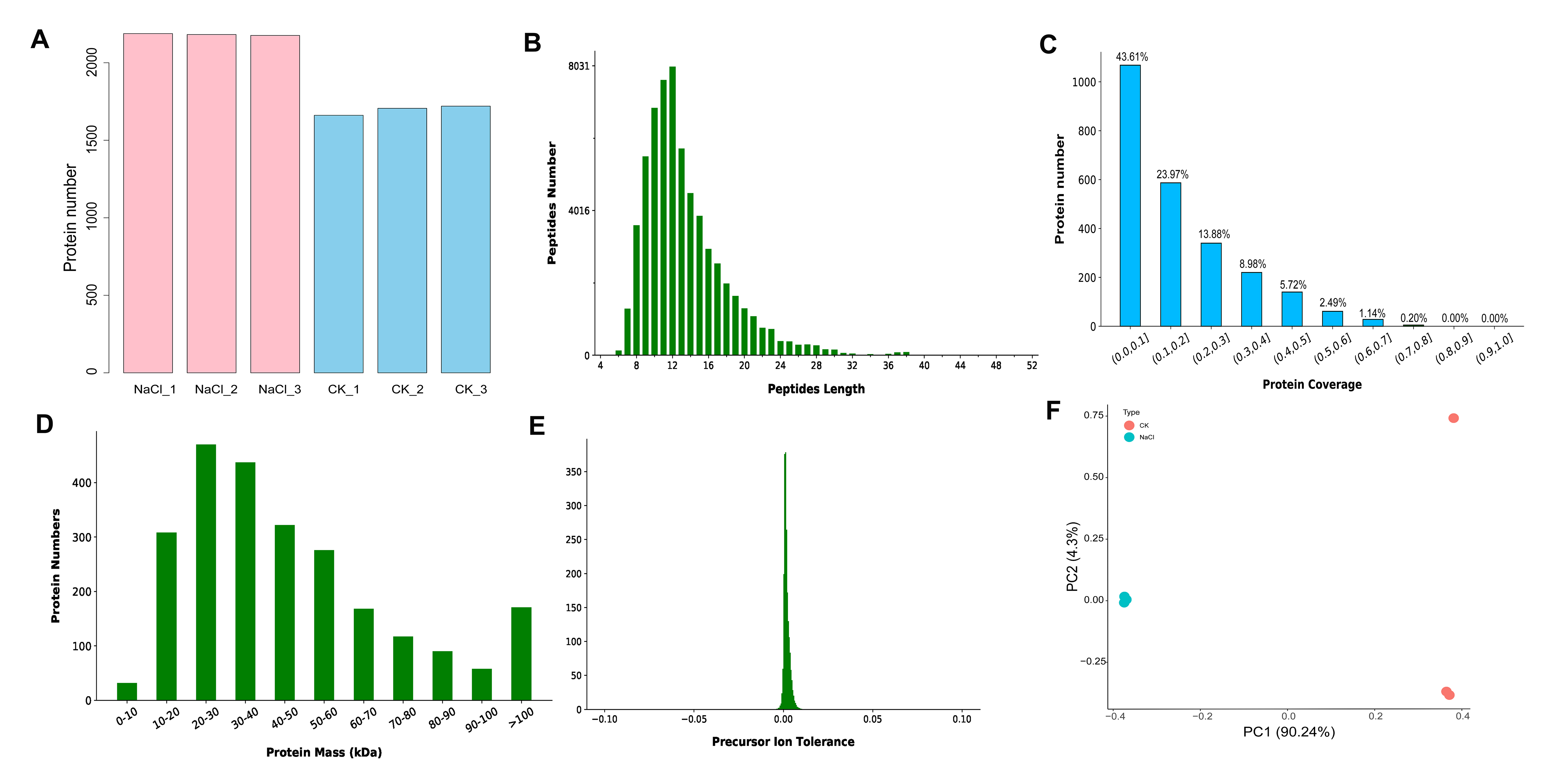

Supplement: Supplementary Figure 1 — Data quality assessment and protein identification. (A) Number of proteins identified in control and NaCl groups. (B) Peptide length distribution and number. (C) Protein coverage distribution and quantity. (D) Protein molecular weight distribution and quantity. (E) Precursor ion mass tolerance distribution. (F) PCA analysis of control group and NaCl treatment group. [file Image_1.JPEG]
